# Risk-stratification of HPV-positive women with low-grade cytology by *FAM19A4/miR124-2* methylation and HPV genotyping

**DOI:** 10.1038/s41416-021-01614-4

**Published:** 2021-11-06

**Authors:** Stèfanie Dick, Frederique J. Vink, Daniëlle A. M. Heideman, Birgit I. Lissenberg-Witte, Chris J. L. M. Meijer, Johannes Berkhof

**Affiliations:** 1 Amsterdam UMC, Vrije Universiteit Amsterdam, Pathology, Cancer Center Amsterdam, Amsterdam, The Netherlands; 2 Amsterdam UMC, Vrije Universiteit Amsterdam, Epidemiology and Data Science, Amsterdam, The Netherlands

**Keywords:** DNA methylation, Diagnostic markers, Cervical cancer, Molecular medicine

## Abstract

**Background:**

The introduction of primary HPV screening has doubled the number of colposcopy referrals because of the direct referral of HPV-positive women with a borderline or mild dyskaryosis (BMD) cytology (ASC-US/LSIL) triage test. Further risk-stratification is warranted to improve the efficiency of HPV-based screening.

**Methods:**

This study evaluated the discriminative power of *FAM19A4/miR124-2* methylation, HPV16/18 genotyping and HPV16/18/31/33/45 genotyping in HPV-positive women with BMD (*n* = 294) in two Dutch screening trials. Absolute CIN3+ risks and colposcopy referrals within one screening round were calculated.

**Results:**

Methylation analysis discriminated well, yielding a CIN3+ risk of 33.1% after a positive result and a CIN3+ risk of 9.8% after a negative result. HPV16/18 and HPV16/18/31/33/45 genotyping resulted in a 27.6% and 24.6% CIN3+ risk after a positive result, and a 13.2% and 9.1% CIN3+ risk after a negative result. Colposcopy referral percentages were 41.2%, 43.2%, and 66.3% for *FAM19A4/miR124-2* methylation, HPV16/18 and HPV16/18/31/33/45 genotyping, respectively. The CIN3+ risk after a negative result could be lowered to 2.8% by combining methylation and extended genotyping, at the expense of a higher referral percentage of 75.5%.

**Conclusion:**

The use of *FAM19A4/miR124-2* methylation and/or HPV genotyping in HPV-positive women with BMD can lead to a substantial reduction in the number of direct colposcopy referrals.

## Background

Human papillomavirus (HPV)-based cervical screening has an increased sensitivity for the detection of high-grade cervical intraepithelial neoplasia (CIN) and cervical cancer compared with cytology-based screening, but has a suboptimal specificity [1, 2] and triage of HPV-positive women is essential to avoid unnecessary colposcopy referrals. However, first evaluations of primary HPV screening in Europe and Australia with triage by cytology and/or HPV16/18 genotyping showed a substantial increase in direct colposcopy referrals compared with cytology-based screening [3, 4].

The Netherlands was the first country that implemented primary HPV screening with cytology triage in the national cervical screening programme in 2017 [5]. HPV-positive women with borderline or mild dyskaryosis (BMD) cytology (comparable with atypical squamous cells of undetermined significance (ASC-US) and low-grade squamous intraepithelial neoplasia (LSIL) in the Bethesda classification) [6] or worse are directly referred for colposcopy. HPV-positive women with normal cytology are re-tested at 6 months and referred for colposcopy in case of BMD cytology or worse [7]. The replacement of the cytological screening programme by primary HPV-based screening with cytology triage has resulted in more clinically relevant findings [1, 2] at the cost of an approximately two-fold increase of colposcopy referrals and ≤CIN1 diagnosis [8, 9]. The increase in colposcopy referrals is mainly caused by the direct referral of women with BMD cytology [9], who often do not harbour high-grade CIN (CIN2/3) [10–12]. Further triage of HPV-positive women with BMD cytology with an objective molecular test that has a higher specificity for high-grade CIN might reduce the number of women referred for colposcopy, while maintaining clinical sensitivity.

HPV16/18 genotyping (partial genotyping) or HPV16/18/31/33/45 genotyping (extended genotyping) has been considered for triaging HPV-positive women with BMD cytology, because CIN3+ risks vary between individual high-risk HPV genotypes [13–15]. An alternative triage strategy is DNA methylation analysis of host-cell genes. Hypermethylation of promotor regions of certain tumour suppressor genes is a crucial step in cervical carcinogenesis [16, 17]. Methylation levels have shown to increase with duration of the HPV infection and with increasing CIN grade, reaching very high levels in cervical cancer [18–21]. *FAM19A4/miR124-2* methylation analysis has shown a high sensitivity for cervical cancer and advanced high-grade CIN (i.e., CIN lesions associated with a HPV infection of at least 5 years) in HPV-positive women [22, 23].

In this study, we evaluate the performance of *FAM19A4/miR124-2* methylation, HPV16/18 genotyping, HPV16/18/31/33/45 genotyping and combinations thereof in HPV-positive women with BMD cytology in two large Dutch population-based screening cohorts.

## Methods

### Study population

This study is a post hoc analysis within the VUSA-Screen trial and POBASCAM trial. HPV-positive women with borderline or mild dyskaryosis (BMD) (VUSA-Screen *n* = 167 and POBASCAM *n* = 192) were selected. Fifty-two samples were excluded due to insufficient leftover material for methylation analysis (VUSA-Screen *n* = 41 and POBASCAM *n* = 11).

#### VUSA-Screen trial

The VUSA-Screen trial is a population-based cervical screening study within the Dutch screening programme with enrolment between October 2003 and August 2005. The VUSA-Screen trial was approved by the Ministry of Public Health (2002/02-WBO; ISBN-10: 90-5549-452-6) and registered in the trial register (NTR215, ISRCTN64621295). The study was designed to evaluate the effectiveness of combined HPV and cytology testing in the Dutch national screening programme. A detailed description of the VUSA-Screen trial has been published before [24]. Cervical scrapes were classified according to the CISOE-A classification, the standard classification system for cytology in the Netherlands, which can be translated into the Bethesda classification system [6]. HPV testing was performed by the HC2 high-risk HPV DNA test (QIAGEN, Hilden, Germany) on cervical scrapes [25]. HPV-positive women with BMD were directly referred for colposcopy. In this *post hoc* analysis, only HPV-positive women with BMD cytology were included.

#### POBASCAM trial

The POBASCAM trial is a population-based cervical screening study within the Dutch screening programme with enrolment between January 1999 and September 2002. The POBASCAM trial was approved by the Medical Ethics Committee of the VU University Medical Center (no 96/103A) and by the Ministry of Public Health (VWS no 328 650) and registered in the trial register (NTR218; ISRCTN20781131). The study was designed to evaluate the effectiveness for CIN2/3 detection of combined HPV and cytology testing in the intervention group compared with sole cytology testing and blinded HPV testing in the control group. A detailed description of the POBASCAM trial has been published before [26, 27]. Cervical scrapes were classified according to the CISOE-A classification. HPV testing was performed on cervical scrapes using the GP5+/6+ PCR-EIA [28]. Women with BMD in the control group were referred for colposcopy if repeat cytology at 6 or 18 months showed BMD or worse. In this post hoc analysis, only HPV-positive women with BMD cytology from the control group were included.

In both studies, histology results were classified as no dysplasia, CIN grade 1, 2, 3, or cervical cancer. Adenocarcinoma in situ was classified as CIN3. Histopathologic follow-up data were collected through the nationwide network and registry of histopathology and cytopathology (PALGA) [29].

### HPV genotyping and *FAM19A4/miR124-2* methylation analysis

HC2-positive samples from the VUSA-Screen trial were tested with GP5+/6+ PCR-EIA. All EIA-positive samples from both trials were typed by reverse line blot (RLB) assay for identification of 14 high-risk HPV types (i.e., 16, 18, 31, 33, 35, 39, 45, 51, 52, 56, 58, 59, 66, and 68) [28]. Samples that were either GP5+/6+ PCR-EIA positive and/or HC2 positive, but negative on RLB for HPV type 16, 18, 31, 33 and 45 were considered negative for HPV16/18/31/33/45.

*FAM19A4/miR124-2* methylation analysis was performed as described previously [30, 31], blinded for cytology and histology outcomes, by quantitative methylation specific PCR (qMSP) on bisulphite converted DNA from cervical scrapes using the QIAsure Methylation Test® (Qiagen, Hilden, Germany) (VUSA-Screen cohort) or its prototype version (POBASCAM cohort). This prototype version was identical regarding to the primers and probes design, PCR conditions and PCR system. All samples had a ß-actin cycle threshold (Ct) value below 26.4 to assure successful bisulphite conversion and sample quality. Thirteen samples had an invalid *FAM19A4/miR124-2* methylation result (VUSA-Screen *n* = 3 and POBASCAM *n* = 10) and were excluded from further analysis. Methylation status was labelled positive if the QIAsure Methylation Test® result exceeded the preset ΔΔCt value threshold for methylation positivity according to the manufacturer’s instructions.

### Data and statistical analysis

Data of the VUSA-Screen trial and POBASCAM trial were pooled and absolute CIN3+ risks were calculated. All CIN3+ detected within one screening round (i.e., up to 4 years) were included. Separate estimates were retrieved for single and combined triage strategies using *FAM19A4/miR124-2* methylation, HPV16/18 genotyping and HPV16/18/31/33/45 genotyping. The performance of five triage strategies was evaluated: (I) *FAM19A4/miR124-2* methylation, (II) HPV16/18 genotyping, (III) HPV16/18/31/33/45 genotyping, (IV) HPV16/18 genotyping combined with *FAM19A4/miR124-2* methylation and (V) HPV16/18/31/33/45 genotyping combined with *FAM19A4/miR124-2* methylation. Strategy (I) was labelled positive if the methylation result was positive. Strategy (II) was labelled positive if HPV 16 and/or 18 was present. Strategy (III) was labelled positive if HPV 16, 18, 31, 33, and/or 45 was present. Strategy (IV) was labelled positive if HPV 16 and/or 18 was present or if the methylation result was positive. Strategy (V) was labelled positive if HPV 16, 18, 31, 33, and/or 45 was present or if the methylation result was positive. Sensitivity and specificity were estimated with Wald 95% confidence intervals (95% CI). Direct referral percentage was calculated as the percentage of positives from each screening strategy and the number of referrals needed to detect one CIN3+ was calculated by dividing the number of screen positives by the number of true positives. We constructed 95% confidence intervals (95% CI) for the risk differences in STATA (version 14.1, StataCorp, College Station, Texas, USA). If the 95% CI did not contain the value 0, the difference was considered significant. All other statistical analysis were performed with SPSS Statistics (version 26, IBM Corp, Armonk, NY, USA).

## Results

The final study population consisted of 294 HPV-positive women with BMD and valid results for HPV genotyping and *FAM19A4/miR124-2* methylation analysis (VUSA-Screen *n* = 123, POBASCAM *n* = 171), in whom 56 CIN3 and 1 cervical carcinoma were detected. The median age of women included was 35.0 years (range: 29–60 years) and median time to CIN3+ detection was 0.7 years (IQR: 0.2–1.3 years). In total, 121/294 women (41.2%) tested methylation positive, 127/294 women (43.2%) tested HPV16/18 positive and 195/294 women (66.3%) tested HPV16/18/31/33/45 positive.

**Fig. 1:**
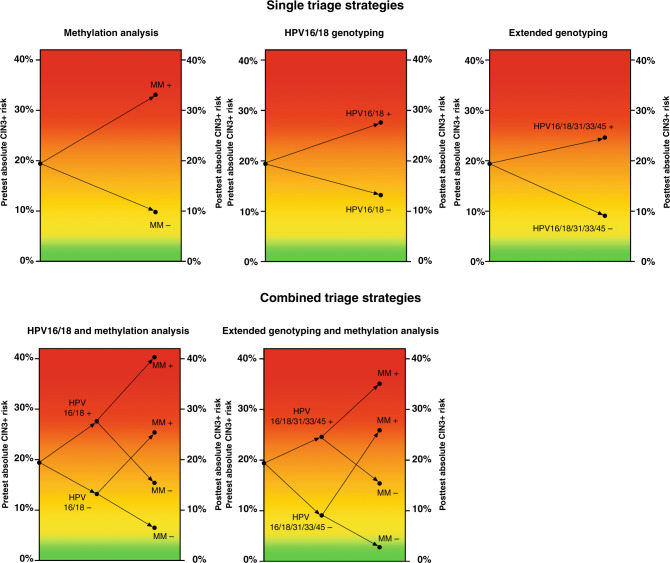
Pre- and post-test CIN3+ risk plots for single and combined triage strategies. Colour legend: green, low CIN3+ risk; orange, intermediate CIN3+ risk; red, high CIN3+ risk; MM+, methylation marker-positive; MM−, methylation marker-negative.

Table 1 shows the absolute CIN3+ risks after triaging HPV-positive women with BMD cytology by *FAM19A4/miR-124* methylation, HPV16/18 genotyping, HPV16/18/31/33/45 genotyping or combinations thereof. Fig. 1 visualises the pre- and post-test CIN3+ risks after application of the single or combined triage strategies. A positive methylation test resulted in the highest absolute CIN3+ risk (33.1%; 95% CI: 24.7–41.4%) and methylation analysis showed the largest risk difference between test-positives and test-negatives (23.2%; 95% CI 13.7–32.7%). The risk difference between HPV16/18-positive and HPV16/18-negative women was 14.4% (95% CI: 5.1–23.7%) and the risk difference between HPV16/18/31/33/45-positive and HPV16/18/31/33/45-negative women was 15.5% (95% CI: 7.2–23.8%). Women who tested negative for *FAM19A4/miR124-2* methylation, HPV16/18 or HPV16/18/31/33/45 had a CIN3+ risk of >9%. Combinations of HPV genotyping and methylation lowered the CIN3+ risk among test-negatives with a CIN3+ risk of 6.5% (95% CI: 1.8–11.1%) among women who were both HPV16/18-negative and *FAM19A4/miR124-2* methylation-negative and a CIN3+ risk of 2.8% (95% CI: 0–6.6%) among women who were both HPV16/18/31/33/45-negative and *FAM19A4/miR124-2* methylation-negative. Supplementary Table 1 reports the absolute CIN3+ risks within the VUSA-Screen trial and POBASCAM trial separately.

**Table 1:** Absolute CIN3+ risks for single and combined triage tests.

Test result	*N*	CIN3+ (*n*)	Absolute CIN3+ risk	95% CI
*Single triage strategies*				
Methylation +	121	40	33.1%	(24.7–41.4%)
Methylation −	173	17	9.8%	(5.4–14.3%)
HPV16/18 +	127	35	27.6%	(19.8–35.3%)
HPV16/18 −	167	22	13.2%	(8.0–18.3%)
HPV16/18/31/33/45 +	195	48	24.6%	(18.6–30.7%)
HPV16/18/31/33/45 −	99	9	9.1%	(3.4–14.8%)
*Combined triage strategies*				
HPV16/18 + and methylation +	62	25	40.3%	(28.1–52.5%)
HPV16/18 + and methylation −	65	10	15.4%	(6.6–24.2%)
HPV16/18 − and methylation +	59	15	25.4%	(14.3–36.5%)
HPV16/18 − and methylation −	108	7	6.5%	(1.8–11.1%)
HPV16/18/31/33/45 + and methylation +	94	33	35.1%	(25.5–44.8%)
HPV16/18/31/33/45 + and methylation −	101	15	14.9%	(7.9–21.8%)
HPV16/18/31/33/45 − and methylation +	27	7	25.9%	(9.4–42.5%)
HPV16/18/31/33/45 − and methylation −	72	2	2.8%	(0–6.6%)

*N,* group total; *n,* number of CIN3+ detected; *CIN3+,* cervical intraepithelial neoplasia grade 3 or worse; *95% CI,* 95% confidence interval; +, positive; −, negative.

Table 2 shows the clinical sensitivity, specificity, colposcopy referral percentages and referrals needed to detect one CIN3+ of five triage strategies. Methylation analysis (strategy I) showed a sensitivity of 70.2% (95% CI: 58.3–82.1%) at a specificity of 65.8% (95% CI: 59.8–71.9%). This strategy resulted in the lowest referral percentage of 41.2% and the lowest number of referrals needed to detect one CIN3. Combined triage with HPV16/18/31/33/45 genotyping and methylation analysis (strategy V) leads to an increased sensitivity of 96.5% (95% CI: 91.7–100%), but specificity decreased to 29.5% (95% CI: 23.7–35.3%) and referral percentage increased to 75.5%.

**Table 2:** Sensitivity, specificity, colposcopy referral percentages and referrals needed to detect one CIN3+ of five triage strategies for HPV-positive women with BMD cytology.

	Triage strategy	Sensitivity (95% CI)		Specificity (95% CI)		Colposcopy referral (%)	Referrals needed to detect one CIN3+
I	Methylation analysis	70.2%	(58.3–82.1%)	65.8%	(59.8–71.9%)	41.2%	3.0
II	HPV16/18 genotyping	61.4%	(48.8–74.0%)	61.2%	(55.0–67.4%)	43.2%	3.6
III	HPV16/18/31/33/45 genotyping	84.2%	(74.7–93.7%)	38.0%	(31.8–44.2%)	66.3%	4.1
IV	HPV16/18 genotyping and/or methylation analysis	87.7%	(79.2–96.2%)	42.6%	(36.3–48.9%)	63.3%	3.7
V	HPV16/18/31/33/45 genotyping and/or methylation analysis	96.5%	(91.7–100%)	29.5%	(23.7–35.3%)	75.5%	4.0

*HPV,* human papillomavirus; *95% CI,* 95% confidence interval.

## Discussion

We evaluated *FAM19A4/miR124-2* methylation, HPV16/18 genotyping (partial genotyping) and HPV16/18/31/33/45 genotyping (extended genotyping) for additional risk-stratification of HPV-positive women with BMD cytology to reduce direct colposcopy referral rates, while maintaining high sensitivity for CIN3+ detection. The choice of additional triage tests depends on the CIN3+ risk, the number of repeat tests and the number of colposcopy referrals that are deemed acceptable.

Of the single triage strategies, *FAM19A4/miR124-2* methylation resulted in the highest CIN3+ risk of 33.1% and the largest risk-difference between test-positives and test-negatives (23.2%). Recently Bonde et al. also showed in a large multicentre cohort that *FAM19A4/miR124-2* methylation detected the very large majority of CIN3 in HPV-positive women with ASC-US/LSIL [32]. In our study, triage with *FAM19A4/miR124-2* methylation led to a referral percentage of 41.2% and a sensitivity of 70.2%. Still, the CIN3+ risk after a negative test for any of the single strategies was >9%. Combined strategies lowered the CIN3+ risk among test-negatives substantially with the lowest CIN3+ risk of 2.8% among women who were both HPV16/18/31/33/45-negative and *FAM19A4/miR124-2* methylation-negative. Despite a high referral percentage of 75.5%, a benefit of this combined strategy is that the CIN3+ risk among triage-negative women seems sufficiently low to recommend return to the regular cervical screening programme.

*FAM19A4/miR124-2* methylation has shown a very high sensitivity for cervical cancer [23] and advanced CIN2/3 lesions (i.e., CIN lesions with a preceding HPV infection of at least 5 years) [22]. Hence, it is assumed that methylation analysis identifies CIN2/3 lesions at highest risk of progression to cervical cancer. This is supported by a recent Finnish study which showed that methylation analysis could serve as a prognostic biomarker to differentiate between regressive and progressive CIN2 lesions [33]. Furthermore, the results of a prospective cohort of women with CIN2/3 with 2 years of follow-up showed that a negative *FAM19A4/miR124-*2 methylation test is associated with high spontaneous regression [34] (Kremer, Dick et al., manuscript in preparation). *FAM19A4/miR124-2* methylation has also prognostic value for development of cervical cancer in the long term. De Strooper et al. found a significantly lower 14-year cervical cancer risk among HPV-positive, *FAM19A4/miR124-2* methylation-negative women compared with HPV-positive, cytology-negative women. Collectively, these data indicate that *FAM19A4/miR124-2* methylation-negative CIN2/3 have high regression rates and low cervical cancer progression risks. As a consequence, methylation analysis may be considered when deciding whether women can be returned to routine screening.

In the new HPV-based screening programme in the Netherlands, the proportion of HPV-positive women who were directly referred for colposcopy because of BMD cytology was 70.3% in year 2019 [35]. The cumulative CIN3+ risk of these women after 2.5 years was 6% [9] and considerably lower than the cumulative CIN3+ risk of 19.4% in our studies after four years of follow-up. This decrease in CIN3+ risk may be related to changes in HPV and cytology test accuracy. A change in the performance of the HPV test is not improbable because the proportion of HPV-positive women was nearly twice as high in the national screening programme as compared with the cohort studies. A change in the performance of cytology can neither be ruled out because cytology in the national programme was conducted with knowledge of HPV status, whereas cytology in the cohort studies was conducted without knowledge of HPV status. Despite these potential screening test generalisability issues, we project that the impact of *FAM19A4/miR124-2* methylation on the number of referrals and detection of CIN3+ will be considerable. Surely, the high colposcopy referral rates and low CIN3+ risk in the national screening programme makes the need for additional triaging of HPV-positive women with BMD even larger than anticipated based on our studies. For the implementation of methylation analysis, applicability and reproducibility of the test among different sample types and DNA extraction methods is essential. Floore et al. evaluated the intra- and inter-laboratory agreement of the *FAM19A4/miR124-2* methylation test with the use of several cervical scrape collection media and several DNA extraction methods and showed a good to excellent intra- and inter-laboratory agreement on the assay and the full workflow [36].

A strength of our study is that we used data of two large population-based HPV DNA screening trials within the Dutch screening programme. As shown in Supplementary Table 1, data of both studies were consistent with comparable CIN3+ risks and therefore results were pooled. Histology endpoints were retrieved from the nationwide network and registry of histo- and cytopathology in the Netherlands (PALGA) [29], which covers all pathology labs in the Netherlands. A limitation is that gynaecological reports were not collected which means that we do not know how many women complied with colposcopy and could potentially lead to an underestimation of CIN3+ risks.

To conclude, the implementation of primary HPV screening with cytology triage in the Netherlands has led to a two-fold increase in direct colposcopy referrals and ≤CIN1 diagnosis, mainly because of the direct referral of women with BMD. Additional risk-stratification of HPV-positive women with BMD by *FAM19A4/miR124-2* methylation could reduce direct colposcopy referral rate with 60%, while retaining high CIN3+ sensitivity. The combination of *FAM19A4/miR124-2* methylation with HPV16/18/31/33/45 genotyping would reduce colposcopy referrals with only 25%, but the low residual CIN3+ risk would obviate the need of short-term follow-up testing.

## Supplementary information

Supplementary Table 1

## Data Availability

The datasets used and/or analysed during the current study are available from the corresponding author on reasonable request.
