# Supplementary material for: Risk-stratification of HPV-positive women with low-grade cytology by FAM19A4/miR124-2 methylation and HPV genotyping
Source: Br J Cancer. 2021 Nov 6;126(2):259–64. doi: 10.1038/s41416-021-01614-4 (PMC8770638; doi:10.1038/s41416-021-01614-4)
Supplement: Supplementary file 1 — Supplementary Table 1 [file 41416_2021_1614_MOESM1_ESM.pdf]

Supplementary Table 1. Absolute CIN3+ risks for single and combined triage tests within the VUSA-Screen trial and POBASCAM trial separately

Test result	N	CIN3+ (n)	Absolute CIN3+ risk	95% CI
**VUSA-Screen study**	**123**	**28**	**22.8%**	**(15.4 - 30.2%)**
Single triage tests				
Methylation +	47	19	40.4%	(26.4 - 54.5%)
Methylation −	76	9	11.8%	(4.6 - 19.1%)
HPV16/18 +	50	17	34.0%	(20.9 - 47.1%)
HPV16/18 −	73	11	15.1%	(6.9 - 23.3%)
HPV 16/18/31/33/45 +	75	23	30.7%	(20.2 - 41.1%)
HPV 16/18/31/33/45 −	48	5	10.4%	(1.8 - 19.0%)
Combined triage tests				
HPV16/18 + and methylation +	24	11	45.8%	(25.9 - 65.8%)
HPV16/18 + and methylation −	26	6	23.1%	(6.9 - 39.3%)
HPV16/18 − and methylation +	23	8	34.8%	(15.3 - 54.2%)
HPV16/18 − and methylation −	50	3	6.0%	(0 - 12.6%)
HPV16/18/31/33/45 + and methylation +	33	15	45.5%	(28.5 - 62.4%)
HPV16/18/31/33/45 + and methylation −	42	8	19.0%	(7.2 - 30.9%)
HPV16/18/31/33/45 − and methylation +	14	4	28.6%	(4.9 - 52.2%)
HPV16/18/31/33/45 − and methylation −	34	1	2.9%	(0 - 8.6%)
**POBASCAM study**	**171**	**29**	**17.0%**	**(11.3 - 22.6%)**
Single triage tests				
Methylation +	74	21	28.4%	(18.1 - 38.7%)
Methylation −	97	8	8.2%	(2.8 - 13.7%)
HPV16/18 +	77	18	23.4%	(13.9 - 32.8%)
HPV16/18 −	94	11	11.7%	(5.2 - 18.2%)
HPV 16/18/31/33/45 +	120	25	20.8%	(13.6 - 28.1%)
HPV 16/18/31/33/45 −	51	4	7.8%	(0.5 - 15.2%)
Combined triage tests				
HPV16/18 + and methylation +	38	14	36.8%	(21.5 - 52.2%)
HPV16/18 + and methylation −	39	4	10.3%	(0.7 - 19.8%)
HPV16/18 − and methylation +	36	7	19.4%	(6.5 - 32.4%)
HPV16/18 − and methylation −	58	4	6.9%	(0.4 - 13.4%)
HPV16/18/31/33/45 + and methylation +	61	18	29.5%	(18.1 - 41.0%)
HPV16/18/31/33/45 + and methylation −	59	7	11.9%	(3.6 - 20.1%)
HPV16/18/31/33/45 − and methylation +	13	3	23.1%	(0.2 - 46.0%)
HPV16/18/31/33/45 − and methylation −	38	1	2.6%	(0 - 7.7%)

Abbreviations: N, group total; n, number of CIN3+ detected; CIN3+, cervical intraepithelial neoplasia grade 3 or worse; 95% CI, 95% confidence interval; +, positive; −, negative
